# A Comprehensive Review of the Manifestation of Cardiovascular Diseases in HIV Patients

**DOI:** 10.7759/cureus.77509

**Published:** 2025-01-15

**Authors:** Yozahandy A Abarca, Bhavya Chadalavada, Jose R Ceron, Boddu Abhinav Sai, Aarzoo Bhatia, Itzel Espinoza, Nidhi L Rao, Razaan Khan, Rimsha Ansar, Zoya Morani

**Affiliations:** 1 Internal Medicine, Escuela de Medicina y Ciencias de la Salud, Tecnológico de Monterrey, Mexico City, MEX; 2 Internal Medicine, Gandhi Medical College, Hyderabad, IND; 3 Medicine, Universidad Popular Autonóma del Estado de Puebla (UPAEP), Puebla, MEX; 4 Medicine, Kamineni Academy of Medical Sciences and Research Centre (KAMSRC), Hyderabad, IND; 5 Infectious Diseases, North Manchester General Hospital, Manchester, GBR; 6 Medicine, Universidad Autónoma de Guadalajara, Guadalajara, MEX; 7 Internal Medicine, K.A.P. Viswanatham Government Medical College, Tiruchirappalli, IND; 8 Medicine, Dow International Medical College, Karachi, PAK; 9 Medicine, Continental Medical College, Lahore, PAK; 10 Medicine, Washington University of Health and Science, San Pedro, BLZ

**Keywords:** cardiovascular diseases, hiv/aids, hiv-associated cardiac conditions, management and prevention of cvd, mechanisms linking hiv to cvd

## Abstract

The increasing lifespan of people living with HIV (PLWH) due to advancements in antiretroviral therapy (ART) has shifted mortality patterns from AIDS-related to non-AIDS-related causes, notably cardiovascular diseases (CVDs). This review investigates how HIV and ART contribute to vascular endothelial dysfunction, myocardial fibrosis, and hypercoagulation, which significantly exacerbate cardiovascular risk. Mechanistic insights include chronic inflammation and immune dysregulation due to persistent HIV infection and ART-specific effects such as protease inhibitors causing dyslipidemia and zidovudine inducing mitochondrial toxicity leading to cardiomyopathy. ART, while lifesaving, has been implicated in promoting subclinical atherosclerosis and increasing the risk of acute myocardial infarction, further highlighting the need for tailored approaches. The manuscript addresses pressing obstacles, including disparities in healthcare access and the lack of standardized cardiovascular screening guidelines specific to PLWH. It emphasizes the integration of advanced imaging techniques and emerging biomarkers, such as coronary artery calcium scoring and soluble ST2, to detect early subclinical cardiovascular abnormalities. The review also identifies challenges in ART selection to balance virologic control and cardiovascular safety. What sets this review apart is its holistic and detailed approach to the intersection of HIV and cardiovascular health. It not only elucidates complex pathophysiological mechanisms but also offers actionable insights into how current clinical guidelines fall short. This manuscript underscores the urgency of implementing proactive cardiovascular screening protocols tailored for PLWH and refining ART regimens to mitigate CVD risks. By addressing these gaps, this work aims to expand our understanding of HIV-related cardiovascular manifestations and provide a foundation for targeted interventions, thereby improving long-term health outcomes for PLWH. This comprehensive perspective is poised to transform clinical practice by fostering greater awareness among physicians and encouraging the development of more effective strategies for managing cardiovascular risks in the HIV population.

## Introduction and background

Breakthroughs in combination antiretroviral therapy (ART) have substantially improved the quality of life for those living with HIV, a far cry from the days when the virus was thought to be fatal. Heart disease is more common in people living with HIV (PLWH) than in the general population [1]. Cardiovascular diseases (CVDs) are twice as common in the HIV-positive population as in the general population [2].

The exact causes of HIV-related heart diseases are still not fully understood, but it is believed that they involve a combination of factors such as vascular dysfunction caused by HIV-induced monocyte activation and cytokine release [3], preexisting conditions, and lifestyle choices, as well as the negative effects of HIV medications on the cardiovascular system [4-7]. Therefore, new research links HIV to coronary artery disease, myocardial infarction, pulmonary hypertension, cardiomyopathies, myocarditis, and heart failure, as well as to thromboembolic events [4-6,8-10]. The current state of knowledge on HIV and CVDs is the subject of this review, which aims to shed light on potential risk factors, treatment choices, and future research directions. Obare et al. investigate the challenges of atherosclerotic CVDs in HIV-positive individuals due to persistent inflammation [11]. Their findings shed light on the complex dynamics of the immune response in HIV infection, showing that while the clinical viral load decreases in ART-using people, the immune response remains continuously dysregulated. The production of the inflammatory cytokines IL-6 and TNF-α occurs when HIV, which is contained inside macrophages, triggers the innate immune response. As a result, they trigger the endothelium surface adhesion molecule synthesis and CD4+ and CD8+ cell recruitment, all of which lead to atheroma development inside the arteries. Additionally, they study how the NLRP3 inflammasome, which increases the production of the important cytokine IL-1β, is regulated in response to HIV infection. They highlight the function of persistent immune activation in a never-ending proatherogenic loop. Furthermore, they investigate how HIV proteins directly influence monocyte and macrophage cholesterol sequestration, which in turn promotes the development of atherosclerotic plaques. Inflammation, cardiovascular risk, and dyslipidemia are already understudied in the PLWH population on ART treatment, and they suggest ways to improve this situation.

Utilizing imaging techniques like computed tomography angiography and positron emission tomography, researchers have recently shown that HIV infection in PLWH is associated with the prevalence of coronary artery calcium and the degree of arterial inflammation, respectively, suggesting a possible correlation between the two conditions and the prognosis of CVDs [12,13].

HIV-associated CVDs have emerged as a leading cause of morbidity among PLWH, driven by chronic inflammation, immune activation, and ART-related metabolic effects. Advances in imaging modalities, such as computed tomography angiography (CTA) and positron emission tomography (PET), are reshaping our understanding of subclinical cardiovascular risks in this population. CTA enables detailed visualization of coronary artery calcium (CAC) and plaque morphology, making it an invaluable tool for early detection of atherosclerosis and identifying high-risk plaques prone to rupture [14]. PET imaging, with radiotracers like 18F-fluorodeoxyglucose, assesses arterial inflammation, offering insights into the systemic inflammatory milieu of HIV-related atherogenesis [15].

Recent research has significantly expanded our understanding of HIV-related CVDs. Chronic immune activation, such as through IL-6 and TNF-α pathways, has been linked to increased plaque vulnerability in PLWH. Furthermore, studies demonstrate that ART, particularly protease inhibitors and integrase inhibitors, may exacerbate endothelial dysfunction and accelerate subclinical atherosclerosis [14]. These insights highlight a paradigm shift from earlier views that focused predominantly on immunosuppression, emphasizing the need for tailored cardiovascular screening strategies for PLWH. By integrating advanced imaging techniques and novel biomarkers, this review underscores the importance of early identification and proactive management of CVD risks in PLWH. This approach aims to bridge existing gaps in current guidelines and foster better long-term outcomes in this vulnerable population.

## Review

Epidemiology

Since HIV was isolated in the early 1980s, it has been detected in 84.2 million people and caused the death of about 40.1 million worldwide. HIV is still a significant health issue, as there are 39 million PLWH globally at the end of 2022 [16]. Since the beginning of the HIV epidemic more than four decades ago, there have been numerous advancements in HIV care and major improvements in its accessibility, leading to longer and healthier lives for PLWH. The rate of mortality due to HIV-related causes and AIDS incidence have significantly gone down because of the increasing knowledge we have on HIV and the extensive use of ART [17]. However, there’s still a high amount of HIV-related morbidity and mortality, which vary depending on geographical areas, socioeconomic status, local healthcare infrastructure, social norms, and education.

CVDs have now become one of the major reasons responsible for HIV-associated morbidity and mortality [18]. Several factors and reasons contribute to the significantly higher incidence and CVD risk among PLWH [7]. PLWH have twice the risk of CVDs than non-HIV-infected individuals, even in the ART era. Additionally, there is regional diversity in the cardiovascular load among PLWH. For example, sub-Saharan Africa is showing the highest prevalence The main underlying cause of HIV-related CVDs is chronic inflammation, which can continue even after the viral load is suppressed by medication [19]. This chronic state of inflammation contributes to endothelial dysfunction and atherosclerosis, thereby elevating the risk of cardiovascular events. Additionally, the mechanisms of HIV infection introduce other risks, including damage from opportunistic infections, autonomic dysfunction leading to arrhythmias, and disruptions to the coagulation cascade. These combined factors exacerbate CVD prevalence and severity in PLWH [20].

Sub-Saharan Africa (SSA) has the highest prevalence of HIV globally, hosting over 70% of the world's HIV-positive population. CVD burden among PLWH in SSA is increasing due to prolonged survival from improved access to ART. For example, studies in rural South Africa demonstrate that HIV-positive individuals on ART exhibit increased carotid intima-media thickness, a marker of subclinical CVDs [21].

Chronic inflammation from HIV infection contributes to endothelial dysfunction, promoting atherosclerosis and heightening risks of myocardial infarction and stroke. Elevated C-reactive protein (CRP) levels and other inflammatory markers have been consistently linked with increased vascular damage and CVD events in PLWH [22]. Specific ART drug classes, such as protease inhibitors (PIs), are strongly associated with dyslipidemia, insulin resistance, and increased CVD risk. For example, dolutegravir-based regimens have been implicated in higher rates of metabolic syndrome, low HDL cholesterol, and elevated triglycerides among PLWH [23]. These metabolic derangements exacerbate endothelial dysfunction and promote atherogenesis. In SSA, socioeconomic barriers such as poverty, inadequate healthcare infrastructure, and low health literacy compound the risks of CVD in PLWH. Many regions face challenges in routine screening for hypertension and dyslipidemia, with significant gaps in access to prevention and management services [24]. Additionally, food insecurity and lack of adherence to lifestyle interventions are prevalent among vulnerable populations [25]. Existing guidelines for managing CVD in PLWH in SSA remain limited, with insufficient integration of HIV-specific factors such as ART regimens into CVD risk prediction models. Addressing these gaps through tailored interventions and robust longitudinal studies is critical to mitigating the dual burden of HIV and CVD [26].

Due to the emergence and successful use of ART targeting HIV, it has now become a chronic disease, and we are witnessing its long-term effects on the human body, including the cardiovascular system. PLWH appear to have an accelerated aging rate, and earlier-onset age-related comorbidities compared to the baseline population [27]. Though ART drugs have saved numerous lives by lowering the risk of opportunistic infection, they come with their own set of risks. We aim to review and summarize the burden of CVD among PLWH through a literature search and review of recent research papers. Understanding the direct effect of the virus on the body, the plethora of opportunistic infections it leads to, the toxicity of the ART, and social determinants of health that PLWH face around the world is critical.

Mechanisms linking HIV to CVDs

The higher incidence of CVDs in PLWH arises from intricate interactions involving HIV-related factors (chronic infection, immune activation, chronic inflammation), as well as standard cardiovascular risk factors (smoking, non-prescription drug use, raised BMI, dyslipidemia, diabetes mellitus, and hypertension), and specific effects of ART [28]. PLWH immune response activates monocytes, macrophages, and CD8 cells, releasing profibrotic and proinflammatory cytokines like IL-6, sTNF-αR1, IL-1β, sTNF-αR2, CCL2, CD163, CD14, and ICAM-1 which cause chronic inflammation, hypercoagulation, collagen overproduction, vascular thrombosis, vascular endothelial dysfunction, hyperlipidemia, left ventricular (LV) dysfunction, and fibrotic remodeling [28]. Continual inflammation can also be maintained by viral co-infections, including hepatitis B virus, cytomegalovirus, and hepatitis C virus [29]. Chronic CMV infection increases memory T cells and strongly induces cytotoxic CD4 cells that move toward the vascular endothelium, promoting early vascular disease and atherosclerosis [30].

HIV also targets CD4 T-cells in the gut mucosa, causing heightened gut permeability to bacteria. Invaders like these set off an inflammatory cascade that opens the SNS and renin-angiotensin-aldosterone channels, leading to free radical generation, endothelial damage, and an upregulation of endothelial angiotensin receptors. This promotes vasospasm, increased vascular permeability, and vascular inflammation which could contribute to CVDs. The viral proteins from HIV, such as glycoprotein-120, trans-activator of transcription, and negative regulatory factor, are also linked to increased inflammation and CVD [17]. A meta-analysis finding indicates that dyslipidemia occurrence with integrase strand transfer inhibitors (INSTIs) is higher than that of protease inhibitors (PIs) and lower than that of non-nucleoside reverse transcriptase inhibitors (NNRTIs). PIs exhibit increased ratios of oxidized LDL to LDL (oxLDL/LDL) and reduced levels of HDL. The incidence of subclinical atherosclerosis is increased by PI use for longer than five years. Patients using the non-retroviral anti-HIV medication didanosine had an increased risk of atherosclerotic coronary artery disease and acute myocardial infarction (AMI), according to the Data Collection on Adverse Events of Anti-HIV Drugs (D:A:D) research [31].

**Figure 1:**
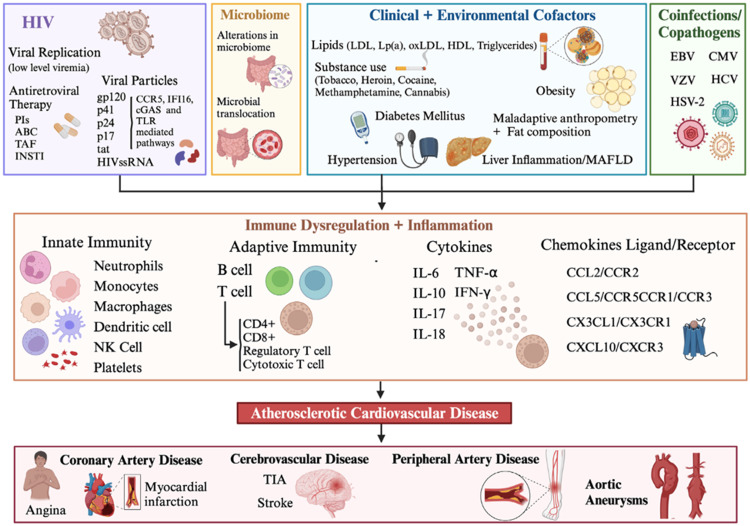
HIV-associated atherosclerosis intersecting pathways. Reproduced under CC BY 4.0. Publisher MDPI [24] CCL: chemokine ligand; ABC: abacavir; CXCL: CXC motif chemokine ligand; CCR: chemokine receptor; CXCR: CXC motif chemokine receptor; CX3CR: C-X3-C motif chemokine receptor; CX3CL: C-X3-C motif chemokine ligand; cGAS: cyclic GMP-AMP synthetase; gp: glycoprotein; IFI16: interferon inducible protein 16; IFN: interferon; HDL: high-density lipoprotein; INSTI: integrase strand transfer inhibitor; IL: interleukin; LDL: low-density lipoprotein; LPS: lipopolysaccharide; Lp(a): lipoprotein (a); NK: natural killer; oxLDL: oxidized low-density lipoprotein; MAFLD: metabolic-associated fatty liver disease; PIs: protease inhibitors; TAF: tenofovir alafenamide; ssRNA: single-stranded ribonucleic acid; TIA: transient ischemic attack; tat: trans-activator of transcription; TLR: toll-like receptor; TNF: tumor necrosis factor.

HIV-associated atherosclerosis results from a combination of factors, including chronic inflammation, immune system dysregulation, and metabolic changes. Persistent inflammation due to HIV and ART leads to endothelial dysfunction, promoting plaque buildup. Additionally, immune imbalances and lipid abnormalities further exacerbate vascular damage and atherosclerosis (Figure 1)[32].

Persistent inflammation in HIV leads to endothelial dysfunction, contributing to atherosclerosis and heightened risks of myocardial infarction and stroke. For example, elevated biomarkers like soluble CD14 (sCD14) and oxidized LDL (oxLDL) correlate with vascular damage and atherogenesis, even in virally suppressed individuals [33]. Mechanistic insights reveal reduced nitric oxide (NO) production and increased pro-inflammatory NF-κB signaling in endothelial cells, exacerbated by the presence of HIV proteins and ART, particularly protease inhibitors (PIs) [34].

Linked to elevated triglycerides, oxidized LDL, and reduced high-density lipoprotein (HDL), PIs are associated with long-term atherosclerotic risks [35]. INSTIs like dolutegravir and bictegravir cause weight gain and metabolic derangements, particularly in women and individuals of African descent [36]. While newer agents like tenofovir alafenamide (TAF) promote weight gain, older agents like tenofovir disoproxil fumarate (TDF) were protective against such effects [36]. Older drugs like didanosine, no longer in widespread use, caused mitochondrial toxicity leading to cardiomyopathy. This historical perspective emphasizes the evolution of ART toward safer profiles, though current regimens still carry risks that must be actively managed [35]. Socioeconomic challenges, such as inadequate access to lipid and hypertension screenings, amplify risks in low-income settings, particularly in sub-Saharan Africa. Integrating routine cardiovascular risk assessment into HIV care remains underdeveloped in such regions [37]. Advanced imaging techniques, like CTA and PET, are critical for the early detection of subclinical CVDs in HIV populations. These modalities guide targeted interventions, including ART optimization and adjunctive therapies like statins to mitigate lipid abnormalities and inflammation [35].

Cardiac conditions associated with HIV

Cardiomyopathy

HIV-associated cardiomyopathy is a stage IV AIDS-defining illness and continues to be a cause of mortality and morbidity in PLWH, despite treatment with ART. The causes and clinical presentation of HIV-induced cardiomyopathy are influenced by the degree of host immunodeficiency. When HIV viral replication is unrestrained, the common causes of HIV-associated cardiomyopathy are myocarditis from direct toxicity of the HIV, opportunistic infections, and malnourishment. On the contrary, in individuals on ART with negligible viral load, the common causes of cardiomyopathy are cardiac autoimmunity, persistent inflammation, and ART toxicity. 

Clinical presentation of HIV-associated cardiomyopathy in high-income countries has altered significantly since ART was started. It has come to notice that subclinical diastolic dysfunction is increasingly more prevalent than systolic dysfunction in individuals with well-controlled HIV [31]. The onset of HIV-associated cardiomyopathy is associated with systemic inflammation and chronic stimulation of the immune system, this may result in diastolic dysfunction and cardiac fibrosis [38].

**Figure 2:**
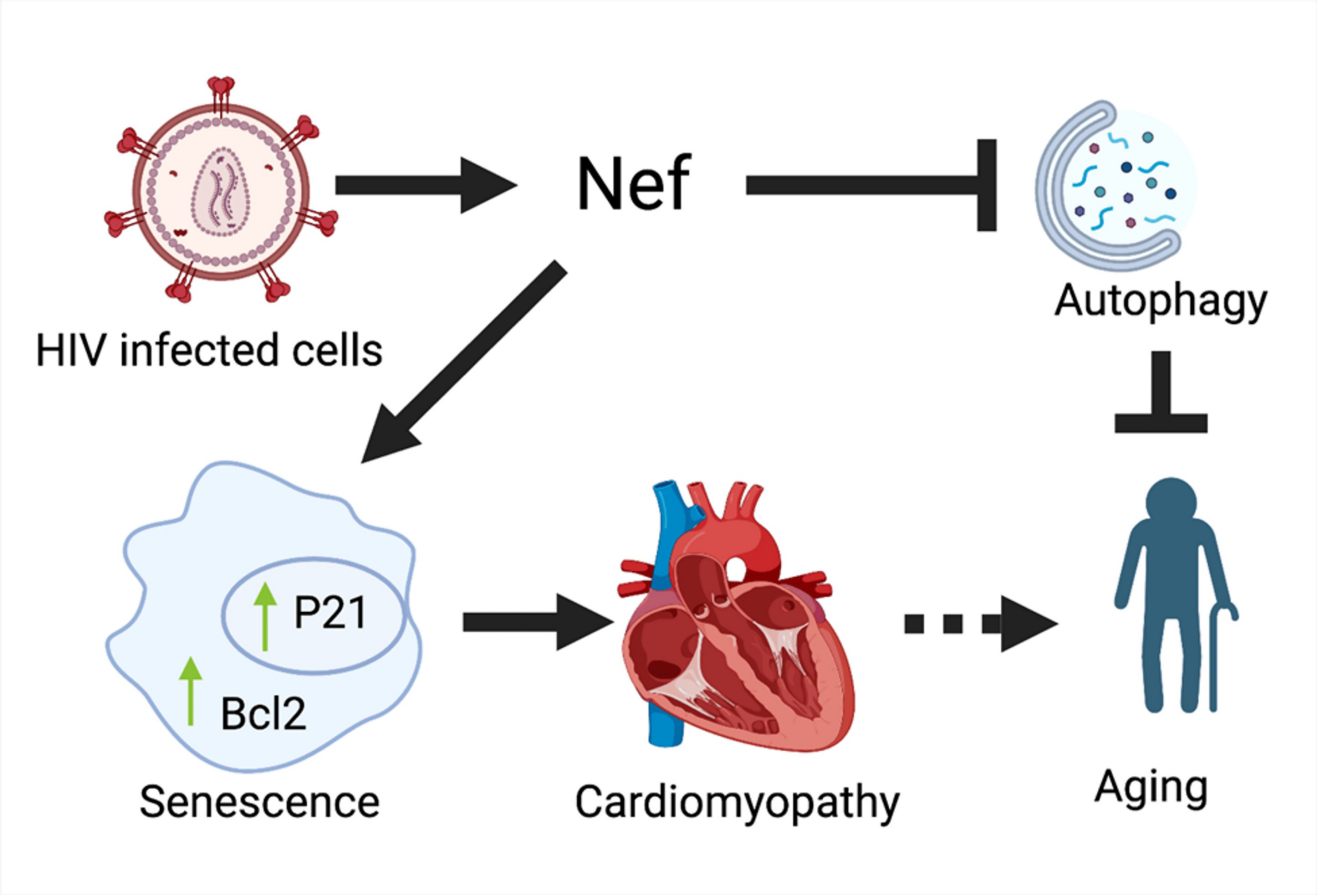
HIV effect on cardiomyopathy Reproduced under CC BY 4.0. Publisher MDPI) [26]

One of the leading causes of death among people living with HIV/AIDS (PLWHA) is cardiovascular disorders that are associated with the virus. The viral load can be reduced with antiretroviral treatment, but it cannot be eradicated entirely. The HIV-1 Nef protein is known to have a role in the virulence and latency of the virus. Heart tissue is one of the tissues where the Nef protein may be found. It is well-established that the Nef protein is important for HIV-1 replication, but how it affects human organ function is less clear. Kondrachuck et al. created a new kind of animal called a Nef-transgenic (Nef-TG) animal that expresses Nef protein in the heart in order to learn more about the effects of Nef on other organs. Our research showed that expression of the Nef gene led to pathological changes in the heart, including impaired cardiac function and increased fibrosis, which in turn caused heart failure and early mortality. Furthermore, we found that cellular autophagy is significantly reduced in the heart tissue of mice lacking Nef-TG. From a mechanistic standpoint, we learned that the Nef protein causes Bcl2 and Beclin-1 to accumulate in the tissue, which may affect the cellular autophagy process. In addition, they found that the expression of Nef causes an upregulation of the cellular senescence marker p21 and the synthesis of β-galactosidase, which is linked with senescence. The data suggest that aging in PLWHA may be facilitated by Nef-mediated inhibition of autophagy and activation of senescence markers as seen in Figure 2 [39].

Myocarditis

HIV-associated myocarditis is described as a condition in which PLWH exhibit a lymphocytic infiltrate of the myocardium along with necrosis and/or degeneration of adjacent myocytes, which is not typical of the ischemic damage linked to coronary artery disease (CAD) [40]. It’s thought that direct invasion of cardiac myocytes in HIV leads to the local production of cytokines and the subsequent infiltration of the myocardium with B-cell clonal growth, resulting in myocarditis [31]. In vitro grown human and rat cardiomyocytes have shown that HIV can enter myocytes directly, unrelated to CCR5 and CXCR4 receptors. Macropinocytosis is one probable theory for this mechanism of invasion [40]. Myocarditis is more common in the latter stages of HIV infection and in PLWH with CD4 counts less than 400 cells/mm^3^ [41]. HIV-associated cardiomyopathy can occur secondary to opportunistic infections, the most common being Cytomegalovirus, Mycobacterium tuberculosis, Herpes simplex, Parvovirus, Coxsackievirus B3, Histoplasma capsulatum, Cryptococcus neoformans and Toxoplasma gondii [42]. According to a major Italian clinical pathological study, cardiotropic viruses, primarily the Coxsackie B3 virus, Epstein Barr virus, and Cytomegalovirus were often co-infected in patients with AIDS and myocarditis [40].

HIV-associated myocarditis and cardiomyopathy can also be attributed to the high levels of circulating immune complexes, autoantibodies, and hypergammaglobulinemia seen in PLWH. High levels of anti-alpha myosin were observed in PLWH with CVDs compared to PLWH without CVDs in a study on PLWH with cardiac-specific antibodies. Additionally, these patients' follow-up survival periods were lower due to higher autoantibody levels [43].

PLWH frequently have micronutrient deficiencies because of diarrhea, wasting syndrome, and intestinal malabsorption, which may lead to the generation of free radicals and cardiac damage. Due to its involvement in other types of dilated cardiomyopathy, selenium is the micronutrient deficiency that has been investigated the most. Selenium deficiency is linked to an increased risk of cardiac injury due to defective immune defenses, phagocyte function, and T cell responsiveness [44]. Whether selenium insufficiency in HF patients is the cause of the disease's origin and progression or only an indicator of a more severe illness is yet unknown.

Regimens based on zidovudine (AZT) may put patients at higher risk for cardiomyopathy. AZT damages mitochondria, blocks mitochondrial DNA polymerase, and results in localized cardiac necrosis. AZT treatment is linked to dose-dependent, reversible myocyte damage in myocytes [45].

Systemic Inflammation and Immune Activation

Systemic inflammation and immune activation are central to the pathophysiology of HIV-associated cardiac conditions. Chronic immune activation, driven by elevated cytokines such as IL-6 and TNF-α, leads to endothelial dysfunction and promotes myocardial fibrosis. This inflammation contributes to both diastolic dysfunction and myocardial stiffness, which are increasingly recognized in well-controlled HIV [33]. Persistent immune activation also accelerates atherosclerosis, linking systemic inflammation to ischemic cardiovascular disease. Interventions such as statins and anti-inflammatory therapies have shown potential in mitigating these inflammatory effects, though their specific application in HIV-related cardiac conditions requires further research [35].

Myocarditis and Cardiomyopathy

HIV-associated myocarditis and cardiomyopathy share overlapping mechanisms, including direct viral effects and immune-mediated myocardial injury. Myocarditis often presents with nonspecific symptoms such as chest pain, arrhythmias, or heart failure, making diagnosis challenging without advanced imaging like cardiac MRI or biopsy. Left untreated, myocarditis can progress to dilated cardiomyopathy characterized by ventricular dilation and systolic dysfunction. The Nef-TG animal model has provided insights into the mechanisms underlying HIV-associated cardiomyopathy, demonstrating that HIV proteins such as Nef induce myocardial damage by disrupting calcium signaling and promoting oxidative stress. While these findings are primarily derived from animal studies, their relevance to human disease lies in the shared pathways of myocardial injury [34].

Selenium deficiency exacerbates these conditions by increasing oxidative stress, although it is not a direct cause of cardiomyopathy. Supplementation may mitigate these effects but is not universally effective, requiring individualized management strategies [36].

ART-Related Toxicity and Nutritional Factors

ART, while lifesaving, introduces significant cardiovascular risks. PIs are associated with dyslipidemia, elevated triglycerides, and oxidized LDL, all of which promote atherogenesis. Older nucleoside reverse transcriptase inhibitors (NRTIs) like zidovudine caused mitochondrial dysfunction leading to cardiomyopathy, though these are less commonly used in modern regimens.

Nutritional deficiencies, particularly selenium and vitamin D, contribute to oxidative stress and immune dysfunction, compounding the cardiac risks associated with HIV. Micronutrient supplementation, while promising, remains adjunctive to comprehensive ART optimization [37].

Diastolic Dysfunction in Well-Controlled HIV

Diastolic dysfunction is increasingly prevalent in HIV patients with effective viral suppression. This condition, driven by myocardial fibrosis and stiffness, often manifests as exertional dyspnea and fatigue. Echocardiographic screening is critical for early detection. Management strategies include lifestyle interventions, such as exercise and weight management, and pharmacological therapies like ACE inhibitors and beta-blockers, which may slow disease progression [35].

Clinical management and preventive strategies

The REPRIEVE trial highlighted the role of pitavastatin in reducing major adverse cardiovascular events among PLWH, demonstrating a 35% risk reduction for atherosclerotic cardiovascular disease (ASCVD) [46]. Studies reveal significant gaps in implementing ASCVD preventive practices, such as lipid management and lifestyle counseling, within HIV clinics. Enhanced guidelines and care models are needed for integration into routine HIV care [47]. Pilot interventions targeting lifestyle changes, such as nutrition and stress management, have shown promise in improving cardiovascular knowledge and perceived risk among midlife women with HIV [48].

A nurse-led strategy using evidence-based algorithms and electronic health records significantly improved blood pressure and cholesterol management in HIV clinics, demonstrating the feasibility of team-based care models [49]. Cardiovascular care for PLWH varies widely across regions due to disparities in resources and healthcare systems, necessitating tailored strategies to address both traditional and HIV-specific risk factors [20].

Diagnosis and management

When myocarditis is suspected, transthoracic echocardiography is commonly the first imaging modality used. Regional wall motion abnormalities, diastolic dysfunction with preserved ejection fraction, and global LV systolic dysfunction can be the most often observed findings at presentation. Dilated ventricles on echocardiogram can distinguish between acute and chronic onset of disease [50]. However, the gold standard test for diagnosis of non-invasive myocarditis is cardiac magnetic resonance (CMR) imaging. Myocarditis is diagnosed on CMR using the 2018 updated Lake Louise Criteria [51]. The most typical patterns on CMR are septal wall intramural rim lesions or sub epicardial non-contiguous lesions in the LV free wall as opposed to LGE in the subendocardial layer seen in ischemic etiologies. The role and frequency of echocardiography in PLWH remain uncertain. Children are recommended to have an echocardiogram at diagnosis, then repeated every few years if asymptomatic; annually if symptomatic [52]. Establishing appropriate screening recommendations should be a top priority due to the rising incidence of subclinical disease and the consequences of delayed diagnosis of cardiomyopathy/heart failure in PLWH [31]. Biomarkers to screen for heart disease in PLWH being studied are B-natriuretic peptide (BNP), soluble ST2 (a biomarker of cardiac stress), and GDF-15, (growth differentiation factor expressed in cardiac injury).

According to WHO guidelines, starting ART is advised regardless of CD4 count. In preclinical disease, early ACE inhibitor and beta-blocker introduction may help prevent the development of severe systolic dysfunction. In populations with micronutrient deficiencies, adjunctive therapy such as carnitine, selenium, and multivitamin supplementation, has been suggested to preserve LV function; however, more research is needed to confirm these claims. The only effective treatments for end-stage HIV-associated cardiomyopathy are mechanical support devices and heart transplantation, although their application remains limited in HIV-affected populations. According to a declaration made by the United Network for Organ Sharing (UNOS), heart transplant consideration should not be denied to asymptomatic PLWH on the basis of their HIV status alone. HIV infection is no longer a contraindication for mechanical support and transplant due to improved outcomes from ART [53].

Transthoracic echocardiography remains a cornerstone for evaluating HIV-associated myocarditis and cardiomyopathy. While regional wall motion abnormalities (RWMA), diastolic dysfunction, and global LV systolic dysfunction can occur in HIV-associated myocarditis, these findings are not unique to this condition and overlap with other cardiomyopathies, such as ischemic or hypertrophic cardiomyopathies. The use of speckle tracking echocardiography enhances diagnostic sensitivity by identifying subtle systolic dysfunction in patients with an otherwise normal echocardiogram [54]. When dilated ventricles are observed, distinguishing acute from chronic disease involves assessing the presence of myocardial edema or fibrosis via CMR, which is considered the gold standard for diagnosing myocarditis. Acute disease often features edema and early gadolinium enhancement, whereas chronic disease is characterized by late gadolinium enhancement without active inflammation [55].

Echocardiography in Children

In children with perinatally acquired HIV, echocardiographic abnormalities, such as diastolic dysfunction and LV hypertrophy, are common even in the absence of clinical symptoms. The recommended frequency of echocardiograms in children reflects the high prevalence of subclinical cardiac involvement, driven by immune activation and ART-associated metabolic changes [56]. Similar strategies are under development for adults, particularly in detecting early diastolic dysfunction via advanced imaging and strain analyses [57].

Cardiac Magnetic Resonance

CMR provides high sensitivity and specificity for myocarditis diagnosis in PLWH. It enables the detection of myocardial edema, hyperemia, and fibrosis through techniques like T2-weighted imaging and late gadolinium enhancement. Studies show that CMR has a sensitivity of 72-85% and specificity exceeding 90% for myocarditis when Lake Louise criteria are applied [55].

Biomarkers in HIV-Associated Cardiac Dysfunction

Cardiac biomarkers like BNP, soluble ST2, and growth differentiation factor-15 (GDF-15) have shown promise in identifying subclinical myocardial dysfunction. Soluble ST2 levels correlate with diastolic dysfunction and fibrosis in PLWH, providing a non-invasive means of risk stratification [58]. However, these biomarkers remain primarily under investigation and are not yet part of routine clinical practice [59].

Clinical Management Strategies

The recommendation to initiate ACE inhibitors and beta-blockers in preclinical cardiac disease among PLWH is extrapolated from general heart failure guidelines. While there is limited HIV-specific evidence, these therapies have demonstrated benefits in reducing fibrosis and improving diastolic function [56]. Potential risks include interactions with ART, particularly with drugs metabolized by cytochrome P450 enzymes, necessitating close monitoring [55].

Nutritional Interventions

Nutritional deficiencies, such as selenium and carnitine, are particularly relevant in resource-limited settings where micronutrient deficiencies exacerbate oxidative stress and myocardial damage. Although supplementation has shown promise in some studies, the evidence remains inconclusive and context-specific [57].

Advanced Interventions

Heart transplantation and mechanical support devices are viable options for PLWH, but access remains limited by stigma, resource constraints, and concerns about long-term outcomes. The United Network for Organ Sharing (UNOS) guidelines permit transplantation in PLWH, but eligibility criteria must account for viral suppression, ART adherence, and absence of opportunistic infections [56].

This comprehensive section integrates the latest evidence, enhancing the understanding of diagnostic tools, biomarkers, and management strategies in HIV-associated cardiac dysfunction.

Sudden Cardiac Death

PLWH have a greater risk of sudden cardiac death (SCD) compared to the general population. The higher risk can be attributed to ventricular arrhythmias caused by multiple factors like myocardial inflammation/fibrosis, presence of cardiovascular risk factors, substance abuse, adverse effects of ART, accelerated atherosclerosis and QT prolongation [60].

Among PLWH, poor control of the virus (as indicated by low CD4 counts and high viral load) is associated with an increased likelihood of ventricular ectopy and ventricular arrhythmias [61]. PLWH with ventricular arrhythmias are at a higher risk for SCD.

A greater incidence of myocardial fibrosis has been observed in PLWH. Different studies evidence the common presence of myocardial fibrosis in PLWH, showing higher indices of myocardial fibrosis on CMR compared to uninfected patients [62]. The exact process through which myocardial fibrosis develops in PLWH remains uncertain, but it could involve the direct impact of HIV on the heart muscle, adverse reactions from antiretroviral therapy, ongoing systemic inflammation, and infectious myocarditis [60].

QT prolongation is common among PLWH; as is known, prolonged QT is a potential cause of dangerous arrhythmias like torsades de pointes. The mechanism of QT prolongation in PLWH is not well established, but it’s possible that the HIV virus may cause abnormalities with the repolarization of the myocardium, as seen in HIV-infected mice [63]. Furthermore, the frequently prescribed medications to PLWH like antibiotics, antifungals, antiprotozoals, antipsychotics/antidepressants, and antiarrhythmics significantly contribute to the prolongation of the QT interval. PIs and NNRTIs, particularly Efavirenz, have been associated with QT interval prolongation and torsades de pointes, so their concomitant use with another QT-prolonging medication should be monitored.

Substance abuse, particularly cocaine and amphetamines, is remarkably more common in PLWH, further elevating the risk of SCD. These substances can induce sympathetic hyperactivity, myocardial ischemia, and nonischemic cardiomyopathy, which can cause life-threatening arrhythmias [60].

Because PLWH are more likely to have typical cardiovascular risk factors like smoking, hypertension, and diabetes, they have been associated with accelerated atherosclerosis [64]. In addition to the conventional risk factors for cardiovascular disease, viral infections that generate persistent systemic inflammation cause early atherosclerosis in PLWH [65]. A meta-analysis looking at SCD in PLWH found that PLWH with HF and lower LVEF had a greater risk of sudden mortality than PLWH with normal LVEF [66]. Early cardiovascular risk assessment is a crucial first step toward multidisciplinary care of PLWH that may lower SCD risks.

HIV and ART have been associated with disruptions in hERG channels, responsible for potassium currents, leading to prolonged QT intervals and increased arrhythmia risks. ART, particularly protease inhibitors and efavirenz, exacerbates this by inhibiting ion channel expression [60]. CD4C/HIV transgenic mouse models demonstrated prolonged cardiac repolarization and altered calcium handling, highlighting direct viral protein effects [67]. HIV contributes to repolarization abnormalities through chronic inflammation and direct myocardial infiltration. Proteins such as Nef and Tat disrupt ion channel expression and calcium signaling [63]. ART, particularly protease inhibitors and NNRTIs, further prolongs QT intervals by inducing oxidative stress and mitochondrial dysfunction [60].

Studies using cardiac MRI show higher myocardial fibrosis prevalence in PLWH (60%) compared to HIV-negative individuals (20%) [39]. Fibrosis mechanisms include systemic inflammation, ART toxicity, and direct viral effects, such as HIV protein-mediated oxidative stress [62].

SCD prevention strategies: ICDs are recommended for PLWH with left ventricular ejection fraction (LVEF) <35% or high arrhythmia risk. Studies show comparable survival outcomes in HIV-positive and HIV-negative ICD recipients [66]. Regular ECG and 24-hour Holter monitoring are critical for early detection of QT prolongation and ventricular ectopy in high-risk patients [34]. Statins, particularly pitavastatin, reduce cardiovascular events in PLWH by 35% [61]. Beta-blockers and ACE inhibitors mitigate fibrosis progression and improve diastolic function in patients with subclinical dysfunction [46]. HIV and ART contribute to cardiac electrophysiological disruptions and fibrosis through inflammatory and direct myocardial mechanisms. Substance interactions amplify risks, necessitating vigilant screening with ECGs and Holter monitoring. SCD prevention strategies, including ICDs and pharmacological interventions, are critical for improving outcomes.

Myocardial Infarction

PLWH are more susceptible to AMI and other CVDs than HIV-negative patients [38]. HIV infection is an independent risk factor for the development of atherosclerotic CVD. PLWH and hypertension are more than twice as likely to experience AMI compared to hypertensive adults without HIV [17]. A systematic review indicated that a high viral load, low CD4 count, high CD8 count, and certain classes of ART are the most significant risk factors for AMI in PLWH [68]. Effective HIV control is crucial for preventing myocardial infarction by maintaining a low viral load and CD4 count ≥500 cells/ml. Keeping HIV viral load below a clinically significant threshold of <400 copies/ml slows atherosclerosis progression.

Early identification of subclinical atherosclerosis could be crucial for prevention of cardiovascular events [38]. One possible factor that needs to be further studied is epicardial adipose tissue (EAT) thickness, as it is higher in PLWH [69]. Carotid intima-media thickness (CIMT) is the measurement of the thickness of the carotid artery wall's intima and media using ultrasonography. PLWH have 0.04 mm thicker CIMTs as compared to the general population according to a meta-analysis [69]. However other studies revealed no significant change in CIMT, hence, further data is needed. An extensive observational cohort study found that increased CIMT is positively associated with age, BMI, years of smoking, and DM2 but negatively associated with HDL-C [29]. Non-contrast-enhanced CT CAC scoring is a simple way to discover and measure coronary calcifications. In the general population, CAC values strongly predict cardiovascular events. CAC predicts cardiovascular events better than CIMT in many studies [70]. PLWH were found to have a higher probability of having higher CAC between the ages of 45 and 50 [38]. Coronary computed tomography angiography (CCTA) distinguishes calcified plaque from non-calcified plaque (NCP) and measures coronary artery stenosis. Compared to CAC and CIMT, CCTA is superior at detecting NCP and predicting CVD [70]. PLWH were also found to have an increased volume of coronary plaque, linked to both CVD risk factors and HIV-related risk variables [38]. According to the MACS study, HIV-positive men, especially those with detectable HIV viremia and higher systemic inflammation, had faster coronary plaque progression than HIV-negative men with well-controlled viral levels and patients with low CD4+ cell counts exhibited higher NCP and coronary stenosis over 50% [71]. Compared to HIV-negative controls, vulnerable plaque was more common in HIV-positive individuals receiving ART according to one study [38]. These findings suggest that the susceptibility of plaques to rupture, rather than the overall extent of atherosclerosis, play a significant role in the development of CAD in PLWH. Another study found that abacavir use increased incidence of noncalcified/mixed plaque suggesting that abacavir may increase cardiovascular risk by increasing atherosclerosis [35,44,70].

PLWH are more likely to experience traditional cardiovascular risk factors [17] and in a major observational cohort study of PLWH, standard cardiovascular risk factors, not HIV-specific variables, were associated with increased carotid atherosclerosis [29]. While traditional risk factors are typically responsible for greater mortality, well-managed HIV infection and healthy lifestyles today have survival rates equivalent to those without HIV [70]. For PLWH to prevent CAD, physicians should optimize traditional risk factor management, start ART early, and maintain regular patient follow-ups.

ART has reduced HIV-related death and disease over time. However, some classes of ART may cause insulin resistance, hyperglycemia, and dyslipidemia which may promote atherosclerosis in PLWH. PIs, NRTIs, and NNRTIs cause hyperlipidemia and contribute to CVD [17]. ART may also thicken the CIMT, cause stenosis of the carotid and coronary arteries, and reduce flow-mediated dilation of vessels. This highlights the complex balance between managing HIV and mitigating cardiovascular risks, emphasizing the need for rational ART selection based on individual cardiovascular risk. Further research is essential to improve our understanding of the effect of ART on cardiovascular health.

Medical stabilization and coronary angiography with stent implantation must be performed within 24 hours of admission in patients with non-ST-elevation myocardial infarction. CCTA and elective percutaneous coronary intervention with stent implantation are options for low-risk non-ST-elevation myocardial infarction patients who have increased blood troponin levels but no recurring chest pain or abnormalities in their electrocardiogram. It is recommended that patients with STEMI have PCI with stent implantation and antiplatelet treatment for PLWH within 12 hours of onset. Stent implantation and percutaneous coronary intervention should be considered for patients who have persistent symptoms for over 12 hours, as well as those who suffer from cardiac arrhythmias, hemodynamic instability, recurrent chest discomfort, or heart failure. It is safe and effective to do coronary artery bypass graft surgery on PLWH with complex CAD who are on anti-rejection therapy and do not have extensive immunosuppression [17].

The impact of co-infections such as hepatitis C (HCV) on AMI risk in PLWH is significant, as HCV co-infection is associated with increased CVD risk and mortality compared to HIV mono-infection. A meta-analysis found that HIV/HCV co-infection significantly increases the risk of CVD, with a pooled hazard ratio of 1.45, and also elevates mortality odds in CVD patients compared to those with only HIV [72]. However, the relationship between HCV and type 1 myocardial infarction (T1MI) is less clear, as one study found no significant association between HCV and increased T1MI risk, although the risk of T1MI increased with age in those with HCV [73]. The inconsistency in carotid intima-media thickness (CIMT) findings may be attributed to varying study methodologies and the lack of randomized controlled trials, which complicates the consensus on HCV's role in atherosclerosis [74]. Regarding CCTA findings, vulnerable plaque characteristics identified in PLWH can guide clinical management by highlighting the need for aggressive cardiovascular risk reduction strategies. The MACS study suggests that increased plaque progression in PLWH is independent of ART use or viral suppression status, emphasizing the need for careful cardiovascular monitoring regardless of ART status. Newer ART regimens, particularly integrase inhibitors, are considered safer for cardiovascular health compared to older protease inhibitors, which have been linked to increased CVD risk. In terms of revascularization, specific considerations for PLWH include potential bleeding risks and ART interactions, necessitating careful perioperative management. Post-procedural modifications in ART regimens may be required to mitigate increased cardiovascular risk, and the utility of statins or antiplatelet therapies tailored to PLWH is supported by recent trials showing the benefits of intensive lipid-lowering therapies in this population [75].

Peripheral artery disease and HIV-associated aortopathy

Without bicuspid aortic valves, a strong hereditary predisposition, or traditional risk factors, aortic aneurysms, and their branches are rare in young people who do not have HIV [76]. Throughout the last four decades, many case reports and series have been reported in both HIV-endemic and non-endemic countries describing the occurrence of aneurysmal and occlusive aortic disease in young HIV-positive persons who are not undergoing antiretroviral medication [77]. Aortic aneurysms are a unique vascular ailment linked with HIV; a recent finding from a European study of people living with HIV who were treated with antiretroviral medication and validated by CT scans provides light on this. There was no correlation between aneurysms and HIV-specific variables such as viral load or CD4+ cell count in this investigation [78]. Evidence from the United States suggests that aortic inflammation may be better estimated using fludeoxyglucose positron emission tomography imaging in asymptomatic ART-treated PLWH compared to healthy controls, and this finding is further supported by the correlation with the monocyte activation marker sCD163 [13]. There is substantial overlap between aortic abnormalities in PLWH and Takayasu arteritis, according to a single French center research study that lasted 15 years and included histology and fludeoxyglucose positron emission tomography-confirmed aortitis. On fludeoxyglucose positron emission tomography, both conditions show extensive inflammation of the main arteries, along with aortic aneurysms and occlusive disease in its main branches. Many arteries, including those that provide blood to the digestive system, the carotid arteries, and the femoral arteries, were involved [79]. There are no obvious regional differences in the clinical presentation of HIV aneurysms, but the largest series are from South Africa. The condition has been reported in several areas. Clinical management necessitates an immediate surgical or endovascular intervention only in cases of severe symptoms or suspicion of imminent rupture since the normal progression of HIV-related aortopathy is not well understood [80].

It is important to include atherosclerotic disease of the aorta in the differential diagnosis and to provide suitable care when it is found in HIV-positive individuals who have traditional risk factors for CVDs [79]. Despite the total number of 19% increased incidence of symptomatic peripheral artery disease in HIV-positive individuals compared to matched controls, no major studies that would change the standard of therapy for HIV-positive individuals have been conducted [81]. There is a lack of consensus in the literature about the relationship between HIV-specific factors and viremia, such as low CD4 levels [82].

Advanced imaging modalities like PET and inflammatory biomarkers such as monocyte activation markers are pivotal in understanding CVDs in PLWH. PET scans, employing tracers like 18F-fluorodeoxyglucose, have demonstrated their utility in detecting arterial inflammation, correlating with subclinical atherosclerosis and systemic immune activation. This inflammation underpins the heightened cardiovascular risks in PLWH, linking immune dysregulation to arterial calcification and plaque formation [14].

Monocyte activation markers, such as soluble CD163 (sCD163), further provide insights into the pro-inflammatory milieu. Elevated levels of sCD163 have been associated with arterial inflammation visualized via PET, supporting its role in advancing atherogenesis in HIV-infected individuals. The integration of PET imaging with biomarker analysis enhances the ability to detect and characterize vascular inflammation, facilitating early identification of at-risk patients [32].

These findings highlight the importance of combining imaging and biomarker approaches to refine cardiovascular risk stratification and inform targeted therapeutic strategies for PLWH. This integrated perspective bridges gaps in traditional diagnostics and underscores the need for proactive management.

Traditional risk factors

Tuberculous Pericardial Disease

Mycobacterium tuberculosis causing tuberculosis (TB) has a significant ratio of deaths from an infectious disorder, particularly more in low to middle-income countries. Although primarily affecting the lungs, M. tuberculosis can infect any organ and often involves the heart. Mortality rates for cardiovascular manifestations such as tuberculous pericarditis (TBP) are near 40% [83]. PLWH are 9-16 times more likely to acquire TB than HIV-negative people [84]. It is noted from reports by the WHO that 8.6% of TB-diagnosed individuals are infected with HIV.

Pericardial compromise encompasses four conditions: (i) Perimyocarditis; (ii) Pericardial effusion; (iii) Acute pericarditis; (iv) Constrictive pericarditis.

TB is responsible for about 38 to 83% of all constrictive pericarditis in TB-endemic areas [83].

Traditional symptoms of acute pericarditis involve pericardial rubbing and stabbing pain in the chest, which are seldom (3-8%) seen in TBP [85]. The presentation is typically insidious, and the usual clinical features are fever, cough, breathlessness, hepatomegaly and chest pain. In constrictive pericarditis, the most frequent clinical signs are raised central venous pressure, hepatomegaly, reduced heart sounds, peripheral edema, ascites, and sinus tachycardia [83]. Without treatment, up to 50% of patients may develop cardiac tamponade, and 85% may die within six months [86]. This highlights the importance of investigating cases with high clinical suspicion in endemic areas.

To diagnose TBP, the tubercle bacillus must be isolated from pericardial fluid or cardiac tissue, or arteries by direct examination or culture. However, isolation is often challenging. Patients with high suspicion of TBP, pericardial effusion, night sweats, weight loss, and fever >38 ºC should undergo pericardiocentesis [83]. A complete approach is needed, involving clinical examination and pericardial fluid tests for ADA levels, IFN-γ levels, and/or M. tuberculosis PCR testing [86]. Strong clinical suspicion and a comprehensive approach that combines echocardiography, CT, and cardiac MRI are needed to diagnose constrictive pericarditis [83]. Reuter et al. created a diagnostic scoring index where a score above 6 yields a sensitivity of 86% and specificity of 85% in diagnosis of TBP. Adenosine deaminase (ADA) testing in pericardial fluid is a sensitive and specific marker for TBP. Sensitivity ranges from 80-100%, and specificity is around 90%, particularly in TB-endemic regions. It is a rapid, inexpensive, and accessible test that serves well in low-resource settings Interferon-gamma (IFN-γ) has high sensitivity (90-100%) and specificity (95-100%) in identifying TBP. It performs better than ADA in terms of diagnostic accuracy but may not be widely available due to higher costs and technical requirements [86].

Emergency drainage is needed for cardiac tamponade. Percutaneous pericardiocentesis, particularly under echocardiographic guidance, is indicated due to the lack of data supporting open drainage [87].

Management and prevention of CVDs in HIV patients

PLWH should talk to their doctor about ways to reduce their risk factors. In order to prevent and cure CVDs, it is essential to quit smoking, limit the use of illegal drugs, and drink to moderate levels. A diet rich in plant-based foods, nuts, healthy grains, and lean proteins like fish and poultry should be the priority for everyone living with HIV. To lose and keep off excess weight, persons who are overweight or obese need to cut down on caloric consumption to less than 1200 to 1400 calories daily and exercise for at least 150 minutes at a moderate level at least five days a week. If a patient has an LDL cholesterol level of 190 mg/dL or higher, is 40 to 75 years old, has diabetes mellitus, has a history of myocardial infarction or stroke, or has a 10-year risk of cardiovascular disease exceeding 10%, pitavastatin or rosuvastatin should be started and the side effects should be monitored [87-89]. Cardiomyopathy, decreased inflammatory markers in the blood, and reduced activation of T cells and monocytes have been associated with pitavastatin and rosuvastatin [90,91]. Because of its interactions with antiretroviral medication, lovastatin and simvastatin should not be given to patients [88].

Clinical evidence supports the use of pitavastatin and rosuvastatin for reducing cardiovascular risk and inflammatory markers in HIV-positive individuals. Studies, such as the REPRIEVE trial, demonstrated that pitavastatin significantly reduces major adverse cardiovascular events (MACE) by 35%. Additionally, it lowers inflammatory markers like soluble CD14 and high-sensitivity C-reactive protein (hsCRP) [46]. Rosuvastatin also shows benefits in reducing carotid intima-media thickness (CIMT) and systemic inflammation, making it a preferred statin for patients with high cardiovascular risk.

PCSK9 inhibitors, such as evolocumab, are highly effective in reducing LDL cholesterol levels, with reductions exceeding 50% in HIV-positive patients. The BEIJERINCK trial highlights evolocumab’s effectiveness and tolerability [92]. However, cost and limited availability in resource-constrained settings pose challenges. To optimize their use, clinicians should prioritize these treatments for individuals with severe hyperlipidemia, refractory to statins, or with LDL levels exceeding 190 mg/dL. Tools like CAC scoring or CIMT measurements can assist in identifying high-risk patients most likely to benefit.

Aspirin is appropriate for HIV-positive individuals with established CVDs or those with a 10-year ASCVD risk exceeding 10%, provided there are no contraindications, such as an increased bleeding risk. Its use can significantly lower the risk of major adverse cardiovascular events in these populations [88].

Certain ART, such as PIs and NNRTIs, can alter the metabolism of antiplatelet agents like clopidogrel and prasugrel by inhibiting cytochrome P450 enzymes. Clopidogrel activation, for example, may be reduced, necessitating alternatives like ticagrelor, unless contraindicated [93]. Regular platelet function testing (e.g., VerifyNow) and close INR monitoring for patients on warfarin are essential for minimizing complications.

Aggressive blood pressure control should be approached with caution in older HIV patients or those with organ dysfunction. A target of <130/80 mmHg is appropriate for high-risk patients, but therapy should be adjusted to avoid hypotension, syncope, or kidney injury [88]. Gradual dose adjustments and regular monitoring of orthostatic blood pressure are essential.

HIV-positive individuals on ART with QT-prolonging effects, such as efavirenz, should undergo baseline ECG monitoring. Combination therapy with other QT-prolonging agents (e.g., macrolides, fluoroquinolones) should be avoided when possible. For high-risk patients, regular QT interval monitoring is essential to prevent life-threatening arrhythmias [63].

Given the elevated plasma concentrations of PCSK9 that correlate with IL-6 in HIV+ individuals, patients who do not achieve a reduction in proprotein convertase subtilisin/kexin type 9 inhibitor, such as evolocumab, may be worth considering for patients with LDL cholesterol who are on statin or statin plus ezetimibe treatment. In the BEIJERINCK trial, which lasted for 24 weeks, evolocumab reduced the LDL concentration to less than 70 mg/dL in 73% of HIV-positive individuals, and medication was well-tolerated [92].

People with CVDs should take aspirin since it does not have any known drug interactions with antiretroviral treatment [94]. The platelet P2Y12 receptor antagonists clopidogrel, prasugrel, and ticagrelor may be restricted or blocked by protease inhibitors and NNRTIs efavirenz and etravirine [93,95]. Hence, platelet activation tests are essential for determining how much ART reduces the side effects of these drugs. Patients using warfarin in addition to antiretroviral medication have a more difficult time attaining therapeutic international normalized ratios. In the first few weeks after stopping HIV treatment, as well as when using warfarin and ART at the same time, constant INR monitoring is crucial. Individuals with HIV should be carefully monitored while using elvitegravir-cobicistat since in certain people, it might raise plasma levels of direct oral anticoagulants such as dabigatran, apixaban, or betrixaban, which could lead to bleeding.

People whose estimated 10-year risk of CVD is 7.5% or higher should begin and maintain blood pressure management with an angiotensin-converting enzyme inhibitor or thiazide diuretic if their systolic or diastolic blood pressure is 140 mmHg or higher, or 90 mmHg or higher, respectively. A blood pressure goal of less than 130/80 mmHg is suitable for those who are at a higher risk of cardiovascular disease, as long as this aim can be reached without inducing hypotension associated with therapy, near-syncope, syncope, or renal impairment [96]. Dolutegravir, an integrase inhibitor, increases metformin plasma concentrations, whereas other protease inhibitors increase thiazolidinedione plasma concentrations, in relation to the treatment of HIV-positive individuals with diabetes. The dose of saxagliptin must be reduced and the dosage of canagliflozin must be increased for patients taking ritonavir or cobicistat [88,97]. Metformin may be given to individuals without renal or hepatic impairment; nonetheless, it is important to monitor their progress for the development of lactic acidosis. Although sulfonylureas pose no health risks to HIV-positive people, using them with ritonavir or nelfinavir may reduce their effectiveness. Insulin has no negative effects on antiviral medication, increases muscle mass, decreases inflammatory TNFα, and is safe for individuals with diabetes who have developed resistance to other drugs. It is also not harmful for those with kidney or liver problems.

Many HIV-positive patients use azole antifungals, macrolide antibiotics, tricyclic antidepressants, and even certain protease inhibitors, all of which lengthen the QT interval on electrocardiograms. These drugs have the potential to cause ventricular tachycardia or fibrillation, which may increase the risk of cardiovascular accidents by around threefold [98].

## Conclusions

In this study, various relevant articles are reviewed to summarize the etiology and pathogenesis of common CVDs among PLWH. By the end of 2022, 39 million people worldwide are living with HIV. Due to multiple developments in HIV care, the mortality has significantly changed but CVDs have become a serious problem among PLWH. This can be due to the disease itself as it is a chronic inflammatory disease and the adverse effects of ART. There is an increased incidence of CVDs like myocarditis, cardiomyopathy, SCD, and subclinical atherosclerosis compared to the general population. This is thought to be a result of chronic inflammation seen in PLWH that damages the cardiac muscles, opportunistic infections, and the effects of ART. By estimating the extent of various risk factors leading to the early onset of CVD as compared to the general population, an attempt should be made to optimize the prevention and treatment of CVDs in PLWH.
